# Dr. Hallvard Lee

**Published:** 1907-02

**Authors:** 


### DEATH OF DR. HALLVARD LEE

Dr. Hallvard Lee, a traveling dentist, died suddenly Sunday morning, Jan. 6, 1907, at 5 o'clock at Prairie Farm, Barron county, from quick pneumonia. The deceased was 45 years of age and came from Norway. He has been in dentistry for the past twenty years. He was formerly located at La Crosse but of late years has traveled in this section of the state, making the small towns and county seats. He did his office work in the laboratory of Dr. W. H. Bailey, this city.

Dr. Lee was well known to many citizens here. He was an upright citizen and unmarried. His remains were interred at Sand Creek, near New Auburn; at that place resides his uncle, Peter Elke, the only relative he has living in this country.

